# Immunometabolism in Obese Asthmatics: Are We There Yet?

**DOI:** 10.3390/nu5093506

**Published:** 2013-09-10

**Authors:** Hashim A. Periyalil, Peter G. Gibson, Lisa G. Wood

**Affiliations:** 1 Priority Research Centre for Asthma and Respiratory Diseases, Faculty of Health, University of Newcastle, Callaghan, NSW 2308, Australia; E-Mails: hashim.A.periyalil@newcastle.edu.au (H.A.P.); lisa.wood@newcastle.edu.au (L.G.W.); 2 Department of Respiratory and Sleep Medicine, Hunter Medical Research Institute, John Hunter Hospital, New Lambton, NSW 2305, Australia

**Keywords:** obesity, immunometabolism, macrophages, mast cells, asthma

## Abstract

Obesity is now recognised as a worldwide epidemic. The recent International Association for the Study of Obesity/International Obesity Taskforce (IASO/IOTF) analysis estimates that approximately 1.0 billion adults are currently overweight and a further 475 million are obese. Obesity has huge psychosocial impact with obese children and adolescents facing discrimination and stigmatization in many areas of their lives leading to body dissatisfaction, low self-esteem and depression. Indeed, obesity is recognised as an important risk factor for the development of several chronic diseases such as hypertension, cancer, asthma and metabolic syndrome. Chronic low grade systemic inflammation is considered as a hallmark of obesity and may possibly explain the link between obesity and chronic disease, in particular the increased incidence, prevalence and severity of asthma in obese individuals. There is now strong evidence for infiltration of immune and inflammatory cells into adipose tissue that drives systemic inflammation and subsequent end organ damage. In addition to adipocytes, the key adipose tissue resident immune cells are macrophages and mast cells. Immunometabolism, as an emerging field of investigation, explores the pivotal role of these immune cells in translating immunological changes to metabolic effects in obesity. Abundance of free fatty acids, along with other inflammatory cytokines shift the balance of metabolic homeostasis to pro-inflammatory status by influencing the development of inflammatory cell lineage, which, further exhibits distinct functional phenotypes. There is emerging evidence for macrophage activation and functional polarization of an anti-inflammatory M_2_ phenotype towards a pro-inflammatory M_1_ phenotype of macrophages in obese adipose tissue. Similarly, studies in both obese humans and murine models reveal the pathognomic presence of an increased number of mast cells in visceral adipose tissue. These suggest a possible contribution of mast cells to the unique metabolome of obese asthma. This review examines proposed multilevel interactions between metabolic and immune systems in obese asthmatics that underlie the negative effects of obesity and may offer significant therapeutic promise.

## 1. Introduction

Obesity is a very large and costly burden [1,2]. There has been an explosive increase worldwide in the incidence of obesity across age groups [3]. Obesity is also emerging as a major risk factor for asthma [4]. Various cross-sectional [5,6,7], as well as longitudinal studies [8], have reported increased incidence [8] and prevalence [7] of asthma and worse asthma control [9] in obese individuals, particularly in women and children [10,11]. Current evidence suggests that obesity could be a predisposing factor for the development of asthma [8]. Although various hypotheses have been proposed to explain the link between obesity and asthma, such as chronically increased systemic inflammation [12,13], the restrictive effect of obesity on lung volumes [14] and common genetic predispositions [15,16], the mechanistic link between obesity and asthma still remains unexplained [17]. Recent studies looking at immunological changes and possible links to their negative metabolic effects (immunometabolism) [18] have added a great deal of insight into the obesity-asthma association.In this perspective, this review will examine proposed effects of immunometabolism in obese asthmatics and suggest future exploration in this aspect, which may offer potential therapeutic promise.

## 2. Immunometabolism in Obesity

**Figure 1:**
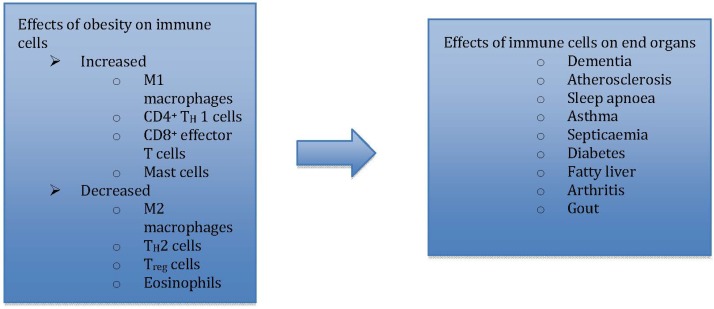
Immunometabolism explores how immune changes are translated to metabolic effects in end organs.

Immunometabolism, as an emerging field of investigation [18], explores the pivotal role of adipose tissue resident immune cells, *i.e*., macrophages [19] and mast cells [20], along with other pro-inflammatory adipo-cytokines, in translating immunological changes to negative metabolic effects in obesity (Figure 1) [21].

### 2.1. Metaflammation in Adipose Tissue

**Figure 2:**
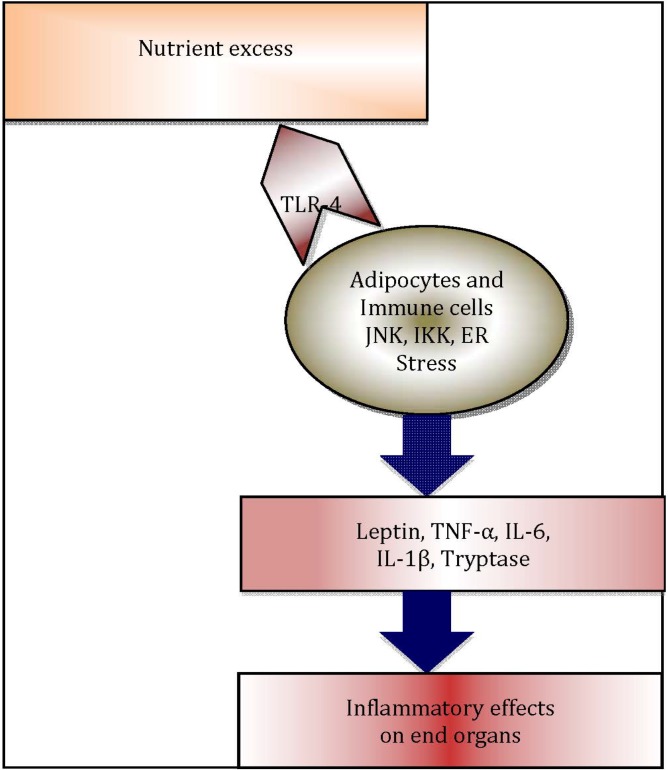
Schematic diagram illustrating inflammatory cascade in obese adipose tissue: Excess nutrients such as free fatty acids trigger endoplasmic reticulum (ER) stress and c-Jun *N* terminal kinase (JNK) and IκB kinase IKK, which are protein kinases that initiate release of inflammatory cytokines. Adipocytes and resident immune cells, notably macrophages and mast cells, secrete inflammatory cytokines, which in turn leads to systemic inflammation and negative end organ effects.

A heightened chronic systemic inflammatory status is now recognised as a hallmark of obesity [21]. Indeed, more recently, this association has been attributed to the distribution of adiposity [22,23,24]. Adipose tissue is now regarded as an important organ regulating metabolic homeostasis [25,26,27,28], in addition to its role as an energy reservoir. Adipose tissue plays a vital role as a buffer in lipid metabolism [29]. The cellular components of adipose tissue, particularly pre-adipocytes, adipocytes, macrophages and fibroblasts undergo hypertrophy as well as hyperplasia [30], in order to buffer the changes in metabolic status. Following the increased intake of dietary lipids, adipose tissue clears the circulation of triacylglycerol (TAG), thus inhibiting release of free fatty acids in to the circulation. However, in the obese state, lipid levels are alarmingly increased and adipose tissue fails to store the excess amount of TAG and free fatty acids. Consequently, the systemic levels of TAG and free fatty acids in adipose tissue increase, leading to “*meta- or para-inflammation*” [21,31]. Free fatty acids can activate innate immune responses through engagement of Toll-like receptor-4 (TLR-4), which in turn initiate a plethora of adipose tissue inflammatory cascades, regulated by endoplasmic reticulum (ER)-stress mediators [32], TLRs [33] and NLRP3 inflammasome-mediated [34,35,36] pathways. These inflammatory processes contribute significantly to increased systemic inflammation in obesity (Figure 2) [26]. Hotamisligil *et al*. were the first to demonstrate the association between obesity-induced adipose tissue inflammation and diabetes, examining the association of increased systemic levels of TNF-α and insulin resistance in obese rodents [37,38]. In addition, in obesity, adipose tissue appears to function as an endocrine organ [39], by secreting adipocytokines and other mediators, contributing to metaflammation [39,40].

### 2.2. Adipokines

Leptin and adiponectin are adipo-cytokines (or adipokines) secretedby adipose tissue and characterised by their pro-inflammatory and anti-inflammatory properties respectively [41,42,43]. Leptin is found at 4 to 6 fold higher concentrations in morbidly obese individuals [44] and the positive correlation between leptin and fat mass, suggests leptin as a vital link between obese adipose tissue and its inflammatory effects [44,45]. Leptin, which is tightly regulated by the *ob* gene, is a plasma protein primarily involved in the regulation of food intake, through its hypothalamic effects [46,47]. Leptin has profound effects on both the innate and adaptive immune systems. In addition to macrophages, leptin exerts its pro-inflammatory effects on dendritic cells, NK cells, T cells, B cells and regulatory T cells through its receptors [48]. Leptin has indirect effects on neutrophils, as they lack leptin receptors [49]. In addition, leptin also activates transcription factors, leading to activation of protein-1 and NF-κB in endothelial cells [50], accelerating atherogenic processes [51] and contributing to development of vascular pathology in obesity [52].

In contrast, adiponectin, an adipokine known to have anti-inflammatory properties, is noted to have an inverse relationship with BMI [53]. The mRNA expression of adiponectin in adipocytes and its systemic levels are decreased in obese individuals [54,55] and its serum level increases with weight loss [56]. Interleukin-6 (IL-6) and tumour necrosis factor-alpha (TNF-α), secreted by macrophages may have a regulatory effect on adiponectin, as evidenced by the *in-vivo* and *in-vitro* interactions between these inflammatory cytokines and adiponectin [57].

Other pro-inflammatory adipokines secreted by adipose tissue include IL-18, tumour necrosis factor (TNF), CC-chemokine ligand 2 (CCL2), CXC-chemokine ligand 5 (CXCL5), resistin, retinol-binding protein 4 (RBP4) and visfatin [39]. C-reactive protein (CRP) and IL-6 are other biomarkers of systemic inflammation, which are increased in obesity [58,59]. CRP was found to be associated with adiposity and cardiovascular risk factors in a cross-sectional study in children aged 10–11 years. However, in this study, Ponderal index (weight/height^3^) was used to measure adiposity, which may not have truly reflected adiposity in children [60]. More longitudinal studies are needed to determine the long-term effects of systemic inflammation on cardiovascular system and the causative relationship in children. In adolescents, a cross-sectional multicentre study (AVENA), designed to evaluate the nutritional status of adolescents, revealed a significantly high CRP in the overweight/obese group, when compared to their normal weight counterpart [61]. In addition, they also noted an increasing trend with other pro-inflammatory markers like TNF-α and IL-6 with overweight/obesity. In adults, the association between increased BMI and increased CRP has been well documented [62,63].

Also, as a result of the hypertrophy and hyperplasia of adipocytes in obesity, perfusion by the existing vasculature is inadequate, leading to apoptotic cell death resulting from tissue hypoxia [64,65,66,67]. The excess uric acid and ATP released by necrotic adipocytes contribute to increased systemic inflammation in obesity.

### 2.3. Macrophage Infiltration of Adipose Tissue

Xu *et al*. [68] and Weisberg *et al*. [69] were the pioneers in studies examining the adipocyte-macrophage association and its pro-inflammatory effects in obesity. Xu *et al*., in their studies involving genetic and high-fat diet-induced obese and diabetic mouse models, found upregulation of macrophage specific genes in the stromal vascular fraction (SVF), which inturn correlated with increased number of macrophages in SVF. These findings were further confirmed by their immunohistochemical analysis [68]. SVF is the extracellular portion of adipose tissue, rich in pre-adipocytes, mesenchymal stem cells, endothelial progenitor cells, T cells, B cells, mast cells and adipose tissue macrophages [70,71]. In a similar study, Weisberg *et al*. found a positive correlation between percentage of F4/80^+^ cells in mice and CD68^+^ cells in humans with increasing adipocyte size, which is a key change occurring to adipocytes in obesity [69].

Intense chemoattractant activity facilitating macrophage migration into adipose tissue has been attributed to inflammasomes [35]. Excess saturated free fatty acids, cholesterol and cellular debris, left after apoptotic cell death, activate NLRP3 inflammasomes to secrete IL-1β [72,73]. IL-1β, along with monocyte chemoattractant protein-1 (MCP-1), facilitates macrophage migration into adipose tissue. These newly recruited macrophages, which surround necrotic adipocytes, have been described as “crown like structures (CLS)” [74,75]. These cells are often described as HAM56^+^ macrophages as they stain positive to a mouse monoclonal antibody, “Macrophage HAM-56” [66,76,77]. These macrophages are activated in the obese state and secrete IL-6, IL-1β, TNF-α and other pro-inflammatory cytokines, which contribute to a low-grade state of chronic systemic inflammation in obesity [78,79]. Moreover, these macrophage-derived cytokines initiate a cycle of adipocyte apoptosis and macrophage recruitment by inhibiting adipocyte differentiation, thus preventing the maturation of pre-adipocytes into adipocytes and further hindering buffering the increased influx of TAG. Furthermore, mature adipocytes continue to hypertrophy, become hypoxic and undergo apoptosis, releasing chemokines and the cycle continues with macrophage recruitment and cytokine production [79].

Cancello *et al*. using immunohistochemical examination of omental and subcutaneous white adipose tissue (WAT) of obese individuals, found that HAM56^+^ macrophages (CLS) were in abundance in omental, when compared with sub-cutaneous adipose tissue. In their correlation analysis, triglycerides appeared to be the best predictor of omental WAT macrophage infiltration. They also demonstrated a significant association between macrophage infiltration in omental WAT and a negative metabolic effect, which was severe hepatic fibro-inflammatory lesions [66]. However, molecular mechanisms linking macrophage accumulation and hepatic lesions were unexplained.

### 2.4. Macrophage Polarisation

Innate responses to pro-inflammatory triggers are characterised by plasticity and diversity in the myelomonocytic differentiation pathway [80,81]. Bacterial moieties such as LPS and T_H_1 cytokines initiate the classical activation of macrophages resulting in a polarising shift towards M1 (pro-inflammatory) macrophage phenotype [82]. M1 macrophages are characterised by their expression of pro-inflammatory cytokines, reactive oxygen species (ROS), inducible nitric oxide synthase (iNOS) and promotion of Th1 response. On the other hand, exposure to IL-4 and IL-13 polarizes macrophages to the M2 (anti-inflammatory) phenotype [82] characterised by increased phagocytosis, scavenging, dampening of inflammation and promotion of tissue remodelling [80,83,84]. In addition, this phenomenon of macrophage polarisation has extended effects on iron [85], lipid [86], glucose [87] and aminoacid metabolism [88].

CD 163 [89], a glycosylated membrane protein expressed exclusively by cells of monocytic lineage (monocytes, macrophages), and its soluble form sCD163 [90], have been extensively studied in the setting of inflammation [91,92,93] as well as obesity and related co-morbidities [94,95]. CD163 has a multitude of functions [96,97,98] and particularly, the uptake of haptoglobin-hemoglobin (Hp-Hb) complexes [99]. Backé *et al*., in their *in vitro* study, found higher expression of CD163 by monocytes and tissue resident macrophages, suggestive of CD163 as a marker of monocyte-macrophage differentiation [100]. The intriguing link between the inflammatory process mediated by macrophages and various clinical effects such as insulin resistance [101,102,103], fatty liver disease [104,105], chronic kidney disease and asthma [25] are still evolving.

### 2.5. Mast Cells

Although macrophages have been studied extensively in trying to understand metaflammation in obesity, other cells have also been examined in this aspect [106]. Mast cells are found in increasing numbers in the WAT of obese humans and mice, when compared to their lean counterparts [107]. Liu *et al*. [108], observed that genetically modified (Kit^W-sh/W-sh^ deficient) mast cell deficient mice, when fed with a high fat and high carbohydrate diet, gained significantly less body weight, when compared to wild type (WT) congenic controls. In addition, they had reduced serum and WAT levels of inflammatory cytokines, chemokines and proteases. A similar observation was noted in mice receiving the mast cell stabilizer, disodium cromoglycate (cromolyn), suggestive of mast cells contributing to systemic inflammation in obesity. In a human study, serum tryptase (ST), which is a marker of mast cell activity [109], was associated with increased BMI and male preponderance [110,111,112].

## 3. Immunometabolism in Obese Asthmatics

Siedell *et al*. [113] and Negri *et al*. [114] in the 1980s were the first to examine the association between obesity and related co-morbidities, such as diabetes, hypertension, asthma, heart diseases, arthritis and cholelithiasis. There is now considerable evidence for a cross-sectional association between obesity and asthma [8,115,116]. This association is more pronounced in obese women [117] when compared to obese men. Furthermore, Brumpton *et al*., in a prospective study [8] examining the effect of fat distribution on asthma, found that abdominal obesity was a risk factor for incident asthma in males and females. Interestingly, in females, the relationship was significant even after adjusting for BMI, suggesting abdominal obesity is an independent determinant of the obesity-asthma association. A similar finding has been reported by Lessard *et al*., in an elegant study categorising obese asthmatics as an unique phenotype based on markers of airway inflammation [11]. In this study, abdominal obesity had a positive relation with serum CRP. Haldar *et al*. reported a significant interaction between atopy status and inflammation in obese adults with asthma [118]. They conducted a cluster analysis to define clinical phenotypes in asthma. Obese, non-atopic women with late onset asthma emerged as a distinct phenotype. Later Moore *et al*., using unsupervised hierarchical cluster analysis, observed similar findings in their “Severe Asthma Research Program” study [119].

### 3.1. Adipokines

Interestingly, various longitudinal studies have shown that obesity precedes the development of asthma, suggestive of a causative role of adipokines [120,121]. There is now evidence for expression of leptin receptors (long (LepRb) and short (LepRa) isoforms) [122,123] and adiponectin receptors (T-cadherin) [124] in bronchial and alveolar epithelial cells in the lung, suggestive of possible effects of adipokines on airway inflammation. Interestingly, leptin can also promote alveolar macrophage activation [125]. However, recent observations by Sideleva *et al*. [126], in a prospective study to examine the association of inflammation in adipose tissue with that of the airways in obese asthmatics, suggest that metabolically active adipose tissue exerts direct effects on airway cells, not involving augmentation of airway inflammation. In summary, the association between leptin and obese asthma still remains inconclusive.

A large study evaluating the role of adipokines in obese asthmatic children, failed to show significant differences in leptin and adiponectin in the obese asthma cohort when compared to their non-obese counterpart [127]. Moreover, the adipokine-asthma association is noted predominantly in prepubertal boys, peripubertal girls, and premenopausal women, suggesting a sex and age dependency [128,129]. Sood *et al*., in their large population based study, found that leptin is associated with incident asthma, particularly in women. Interestingly, the relationship between BMI and asthma remained unchanged even after adjusting for serum leptin concentration. This is suggestive of a partial role for leptin in obesity asthma association as observed in previous studies [130,131,132].

Adiponectin may have a possible protective role in obese asthmatics, particularly in women [129,133,134]. However, a plausible effect of low adiponectin on airway inflammation has been most convincingly shown in mouse models rather than in human studies [135,136], which are complicated by factors such as degree of adiposity, disease severity, medication use and sex hormones. Sood *et al*. [137,138] has demonstrated the complexities of the adipokine-asthma relationship with two large population based studies. A cross sectional analysis showed an independent protective effect of high serum adiponectin level against prevalence of current asthma in premenopausal obese women. However, the relationship between high BMI and current asthma in women remained unchanged after adjusting for serum adiponectin levels. Furthermore, in a recent cross-sectional and longitudinal analysis of a large dataset of obese asthmatics, higher adiponectin levels were associated with worse clinical outcome in men, measured by self-reported symptoms and longitudinal decline in FEV1 [139]. Possible interactions with sex hormones may explain the gender specific effect on adipokines [140,141].

### 3.2. Macrophages

Recently, various cross sectional [101,142] and longitudinal [94] studies have reported a pivotal role for macrophage activation in obesity associated type-2 diabetes. Furthermore, these studies found sCD163 as a significant predictor of insulin resistance, a characteristic feature of obesity associated type-2 diabetes. However, a potential role of macrophage activation in the obese asthma association still needs to be explored. Dixon *et al*., in a unique study, examining the association between adipose tissue inflammation and asthma in obese individuals, found significantly increased macrophage infiltration of visceral adipose tissue (*p* < 0.01) and a similar trend in subcutaneous adipose tissue. However they found an inverse relationship between adipose tissue and airway macrophage activation in obese asthmatics [25]. Further studies exploring the potential role of macrophage activation in the obese asthma association may enable us to have a greater understanding of negative effects of obesity on clinical aspects of asthma.

### 3.3. Mast Cells

There are emerging data to suggest a distinctive role for mast cells in the inflammometry of the obesity-asthma relationship [143], adding a different perspective to the known effects of mast cells in asthma [106,144,145,146,147,148]. The pathognomic presence of increased number of mast cells in airway smooth muscle in asthma is indicative of vital role of mast cells in the pathophysiology of asthma [144]. Chronic mast cell activation results in release of histamine, prostaglandin (PG) D_2_ and leukotriene (LT) C_4_, in turn leading to bronchoconstriction, mucosal oedema (airway inflammation) and mucus hypersecretion, which are fundamental abnormalities in asthma. In addition, mast cell mediators like histamine, tryptase, leukotriene (LT)-D_4_, and TGF-β contribute to smooth muscle cell proliferation [149,150] and potentiate airway hyper-responsiveness [151] by secreting cytokines following activation. Moreover, increased numbers of mast cells in the airway smooth muscle [152] and their degranulation in fatal asthma [147,153] is suggestive of their effect on asthma severity. Nevertheless, the role of mast cells in obese asthma still remains inconclusive.

Fenger *et al*., in a population based study, showed increasing levels of serum tryptase with increasing BMI. However, the increase in tryptase level did not appear to be a determining factor in the association between BMI and symptoms of allergic respiratory disease [112]. In addition, a study in children to determine the relationship between serum tryptase, BMI, sex, ethnicity and atopy, failed to show a significant association between serum tryptase and atopy [110]. This was comparable to findings of a similar study in adults by Gonzalez *et al*. [111]. Further studies are warranted to examine the role of mast cells in obesity-asthma association.

## 4. Effect of Immunometabolism on Airway Inflammation in Obese Asthmatics

Airway inflammation in obese asthmatics is largely determined by the activation of the innate immune system [154]. Airway inflammation in obesity has been assessed using exhaled nitric oxide, induced sputum, and exhaled breath condensate. Various studies using exhaled nitric oxide as a measure of eosinophilic airway inflammation [155], found no significant difference in measurements in obese and non-obese asthmatics [156,157]. Furthermore, studies involving induced sputum cell counts in obese and non-obese asthmatics revealed an inverse relationship between BMI [158] and waist circumference [11] with sputum eosinophilia. These findings are suggestive of obese asthmatics as a distinct phenotype characterised by non-eosinophilic airway inflammation [10,159,160].

Toll like receptors [161] play a vital role in activation of innate immune system by recognising triggers, such as microbial pathogens, macrophages, polymorphonuclear leukocytes and mast cells through their receptors. They, in turn, initiate a cascade of inflammatory and immune responses resulting in negative metabolic effects (Figure 2). Interestingly, there is also increasing evidence for TLR4 activation by dietary fatty acids [33,59]. A heightened TLR-4 [162], TLR-2 [162] and NF-κB [163] mediated immune response was noted following a high-fat meal in obese men and was characterised by increased systemic inflammation [164]. In another model of lipid-induced inflammation, oxidized low-density lipoprotein (LDL) was shown to initiate the NF-κB signaling pathway [165]. Moreover, in healthy adults, a high fat meal was found to be associated with elevated systemic levels of triglycerides along with an increase in exhaled nitric oxide (eNO), which is a measure of airway inflammation, although this study failed to show any effects of high fat meal on CRP and lung function [166]. In addition, Wood *et al*., using an acute fat challenge model in obese asthmatics [167], have convincingly shown that increased airway inflammation following dietary fat is primarilyTLR-4 mediated, although other innate immune receptors may also be involved in this phenomenon [168]. Lipopolysaccharides are the key nutritional elements having significant effects on macrophago-centric immune system activation in obesity. However, it is likely that other dietary components also contribute to airway inflammation. Wood *et al*., in a large randomized control trial have shown the positive effects of anti-oxidant diet on asthma control [169]. Also, in an anti-oxidant withdrawal study, neutrophils were found to be increased when the diet was depleted of antioxidants [170].

Contrary to previous thoughts that airway inflammation is not a determinant of the obesity-asthma association [11,171], there is emerging evidence for neutrophil dominance in airway inflammation in obese asthmatics [172], particularly in females [118,173]. Scott *et al*., in a cross-sectional study, showed that obesity is associated with an increase in neutrophilic airway inflammation [174]. This finding has important clinical implications, as there is mounting evidence to suggest that neutrophilic airway inflammation may account for the refractoriness of obese asthmatics to conventional treatment of asthma [173,175,176], based on inhaled corticosteroids. In the study by Scott *et al*. [177], systemic inflammation was also found to be significantly high in obese asthmatics, as reflected by elevated levels of CRP (*p* ≤ 0.0001), leptin (*p* ≤ 0.001) and IL-6 (*p* = 0.013). Interestingly, a positive association was also found between neutrophilic airway inflammation, and circulating CRP (*r*_s_ = 0.283, *p* = 0.005) and IL-6 (*r*_s_ = 0.284, *p* = 0.005) levels. Gender analysis revealed that the increase in sputum neutrophil percentages in obese asthmatics was driven by females. The mechanism behind the gender effect on airway neutrophilia is unknown. As women have a higher proportion of body fat, it is likely that the effect of adipose tissue may be more pronounced in women than men [178]. Indeed, leptin correlates more strongly with adiposity in women [179,180]. Leptin promotes Th1-cell differentiation and increases activation of neutrophils via TNF-α [43] and hence may contribute to increased airway neutrophilia in females. Alternatively, as obese women have increased levels of oestrogen, which is an independent risk factor for asthma, the effect may be due to an interaction between oestrogen, adipokines and airway inflammation [181]. This is an important area for future research.

The role of alveolar macrophages in obese asthma also requires further investigation. Fitzpatrick *et al*. in a cross sectional study, to examine the role of alveolar macrophages in children with poorly controlled asthma, found impaired alveolar phagocytosis and increased apoptosis [182]. These findings might be suggestive of a potential role for macrophages in increased airway inflammation in childhood severe asthma [183]. Recently, Lugogo *et al*. [184] reported an altered response of alveolar macrophages in obese asthmatics when compared to their non-obese counterpart. A heightened response of alveolar macrophages was noted when bronchoalveolar lavage fluid from obese asthmatics was pre-treated with leptin and further exposed to bacterial lipopolysaccharide (LPS). Furthermore, in obese asthmatics, leptin alone was found to induce macrophages to produce proinflammatory cytokines [185]. This is suggestive of alveolar macrophages potentiating inflammation of the airways and also as an effector agent between increased adipocytokines and airway inflammation in obese asthmatics. Ruby *et al*., comparing efferocytosis by alveolar macrophages and peripheral blood monocytes, found a significant reduction in the efferocytotic property of alveolar macrophages in obese asthmatics [186]. Furthermore, this finding was associated with decreased steroid responsiveness and markers of the M2 macrophage (anti-inflammatory) phenotype, thus highlighting the possible effects of airway macrophages on clinical aspects of obese asthma. Further studies are needed to investigate the potential role of macrophages in modulating airway inflammation in obese asthmatics.

With regard to the role of mast cells in airway inflammation, no human data is available at present. However, in a murine model, high fat-induced obese Balb/c-OVA sensitized mice had greater airway inflammation, characterised by BAL mast cells, which correlated with tachykinin substance P (SP) [187]. This suggests that mast cells may be a possible link between obesity and asthma [188].

## 5. Therapeutic Possibilities

In obese asthma, conventional treatment modalities are not as effective in achieving asthma control and quality of life [9,176,189]. Indeed, the intricacies of the complex signalling networks connecting immune changes to metabolic effects impose a significant challenge in determining the mediators and effector cells where probable intervention would benefit. Various key transcriptional regulators have been studied in this aspect. A strategic approach involving modulation of mediators and effector cells [190,191,192] may be beneficial, rather than attenuation of inflammatory cells, in view of the potential side effects.

The three peroxisome proliferator activated receptors (PPAR-α, PPAR-β, PPAR-γ) are known to regulate lipid and glucose homeostasis in adipocytes, liver and muscle [193]. PPARs serve as nuclear receptors and by induction of other regulatory pathways can indirectly modulate cholesterol metabolism [194] and inflammatory responses [195]. These receptors, particularly PPAR-γ, were reported to induce a shift to oxidative metabolism [19], which in turn is a significant inducer of alternative activation of macrophages [196]. PPAR-γ agonists are noted to have anti-TNFα effects in adipocytes and thus improve insulin resistance [197]. On the other hand, Yamauchi *et al*. [198], in a mouse study, reported that PPAR-γ promotes fat storage by inducing triglyceride accumulation. Furthermore, inactivation of PPAR-γ receptors by deletion [199], antagonist activity [200] and deficiency [201] have shown favorable effects on inflammation in mice studies. This evidence is suggestive of tissue specific diverse actions of PPAR-γ. Further studies are needed to elucidate PPAR-γ targeted treatments options.

In contrast, PPAR-δ agonists have received much attention recently, as they have been shown to inhibit macrophage-mediated inflammation [195,202], lipoprotein lipase activity and activate fatty acid uptake and β oxidation [202]. In addition, PPAR-α agonists like fibrates, Aleglitezar and GFT 505, with lipid-lowering properties [203], have been extensively studied and are noted to have limited efficacy and potential side effects. Currently, a highly specific PPAR-α agonist, K-877, is undergoing clinical trials and demonstrates a low incidence of adverse events [204]. Metabolomics, a method of comprehensive measurement of small molecule metabolites and biomarkers in biological fluids [205], is an emerging and promising technique to understand the role of PPARs and possibly utilise them as drug targets [206].

CD-163, with its unique expression pattern and the ability to transfer substances across the plasma membrane due to its endocytic receptor property, makes it a prospective target for intervention [207]. However, possible binding of CD163 has directed therapeutics to the soluble form [208], which is particularly high in serum under inflammatory conditions. This is a potential challenge, when targeting macrophages as a therapeutic option.

Since mast cells have been implicated in obesity [209] and are a crucial link for expression of obesity-related stress effects on end organs [210], one would expect mast cells to have a key regulatory effect on the obese-asthma association. However to date there is lack of evidence to support the therapeutic use of mast cell stabilisers in obese asthma [211,212,213].

In view of the recent therapeutic advances in formulating steroid sparing therapeutic strategies for obesity and related comorbidities, particularly asthma, it is possible that in future, we may be able to develop specific targeted treatment modalities, not only to improve outcomes, but also to reduce the side effects of inappropriate treatment.

## 6. Conclusions

Although significant progress has been made over the last decade in understanding obesity-induced metabolic and end organ effects, much remains to be discovered for a dogmatic change in the understanding of obesity-asthma association. In this review, we have examined various proposed multilevel interactions between metabolic and immune systems in obese asthmatics. There is now evidence for immunological-metabolic interaction, mediated by reciprocal expression of common receptors, ligands and signalling networks resulting in metaflammation. Interestingly, there seems to be sharing of pattern recognition receptors for host defence during sepsis and metabolic stress in obesity, in turn leading to increased systemic inflammation. In spite of convincing evidence for macrophage and mast cell activation in obesity, it still remains unclear how they might contribute to the intriguing obesity-asthma pathogenesis. The effects of asthma, when occurring in conjunction with already altered airway inflammation and lung mechanics due to obesity, is more likely to possess significant challenges, particularly from a therapeutic perspective. Indeed, obese asthmatics have been recognised as a distinct phenotype. However, future studies are warranted for further categorisation of obese asthmatics according to age, sex and distribution of adiposity, which may enable us to develop targeted weight loss strategies and therapeutic options for obesity related metabolic diseases.
